# Surgery

**Published:** 1892-09

**Authors:** John Parmenter

**Affiliations:** Professor of Anatomy and Assistant Professor of Clinical Surgery, Buffalo University, Medical College


### Progress in Medical Science.

### SURGERY.

Conducted by JOHN PARMENTER, M. D.,

Professor of Anatomy and Assistant Professor of Clinical Surgery, Buffalo University, Medical College.

### BRUISES OF THE BRAIN.

Sir Wm. Savory, (*Lancet*, July 2, 1892,) in a lecture on Bruises of Internal Organs, says of bruises of the brain: "Bruises of the brain are, I believe, far more common than is usually supposed, and worthy of much more attention than they have hitherto received. In works on surgery, contusion of the brain obtains a passing notice and occasionally, as at St. George's Hospital, a specimen to show contusion of the brain is to be found in our museums, but in practice it is for the most part included in the more vague and comprehensive condition of concussion. The relation of contusion to concussion of the brain presents some points of great interest. On what happens in a case of concussion pure and simple, we are not quite clear; certainly, we are not agreed, whether it be due to some sudden and temporary change in the minute vessels in the way of spasm, or whether — which I venture to think is more probable — it is due to some more subtle and profound molecular disturbance of the brain substance itself. As a matter of fact, we know that cases of simple concussion severe enough to prove fatal are so very seldom seen that some even doubt whether they ever actually occur. That, however, concussion of itself, pure and simple, may prove fatal, and that more frequently, when death from injury of the brain is attributed to other causes, it is really due to concussion I have no doubt, and the facts and arguments for this belief I have given elsewhere. So far, however, as the brain itself is concerned, the chief changes that are associated with severe concussion are contusion or bruising. In the worst instances this is accompanied by laceration of the brain substance and copious hemorrhage, so that the blood collects in a distinct mass, but many cases occur, short of these, in which there is neither visible laceration nor blood-clots, but simply a bruised condition of the cerebral substance, and this, perhaps, in a situation opposite to the part struck.

When, in such an instance, a section of the brain is made, the white or grey matter, as the case may be, exhibits a patch or patches, pink or red from bloodstain ; and when these are more minutely examined, especially after water has been allowed to flow freely over the surface, numerous minute points or specks of blood appear scattered over the stained area. In these places the substance of the brain show the effects of bruising. There is here some rupture of substance and some extravasation of blood, but no visible laceration, and nothing which would be called hemorrhage. There cannot, I think, be any reasonable doubt that this condition frequently occurs as the result of injury to the head short of any graver effect, and that such cases usually terminate in recovery.

Under the head of Concussion of the Brain, cases are described in which recovery is not rapid and direct, but more prolonged and circuitous, in which the patient passes days and even weeks in a state of partial unconsciousness or constant drowsiness, with sometimes fits of restlessness, from which, perhaps, he may be temporarily aroused to make short replies to simple questions, but into which he immediately relapses when left to himself. Then, as consciousness is gradually restored, the mind remains in a dull and lethargic state, interference of any kind is often resented, and the temper is sometimes strangely altered for the worse. Usually headache, more or less severe, is complained of. But the bodily functions in general in this stage are performed fairly well, and there is no fever or sign of serious disturbance in any other organ than the brain. In such cases many months often elapse before complete recovery is assured, and during this period its progress is easily interrupted by any attempts to exert the mind. Patients in this state complain that they cannot attend to business, that they are incapable of sustained mental effort, that they have lost the power of self-control, that their senses of sight, hearing, and taste are strangely disordered, and so on."

---

### THE RESULTS OF REMOVAL OF THE UTERINE APPENDAGES.

Dr. Wharton Sinkler (*University Medical Magazine*,) sums up a timely article upon the effects of removal of the uterine appendages :

" The remote effects of removal of the ovaries and tubes upon the general health are, as a rule, to improve nutrition and to better strength, especially if the operation has been done for diseased ovaries or pus tubes ; that excessive gain of flesh is rare, and that change of voice, growth of hair on the face, and loss of feminine characteristics do not occur; that the sexual appetite is seldom changed within two or three years after the operation, but after several years it becomes lessened; that it is often the case that after this operation patients are more nervous than formerly, and mental disturbances of various forms, insanity, and epilepsy, not infrequently follow it; that the influence of the operation is sometimes good upon the insanity and epilepsy which are associated with severe dysmenorrhea, or occur periodically at the menstrual epochs; but when the insanity is constant, although it may be aggravated at the monthly periods, removal of the appendages is of no benefit. Hystero-epilepsy is seldom permanently cured by the operation. Prolonged after-treatment is generally necessary to relieve such cases.

"Local pain is often not relieved by the operation.

"Certain cases of neurasthenia which are associated with dysmenorrhea, or with structural changes of the ovaries, are cured by the operation; nevertheless, no such case should be subjected to the operation, without beforehand having the benefit of prolonged and patient treatment. It is unjustifiable to remove the ovaries and tubes in cases of neurasthenia, hysteria, etc., when these organs are healthy."

---

### THE PRESENT STATUS OF THE SURGERY OF THE VERMIFORM APPENDIX.

WYETH (*International Journal of Surgery*, July, 1892,) gives a clear and concise statement upon this subject. He describes the first case in which an operation was done after a diagnosis of perforation of the appendix had been made, and which resulted in recovery. The anatomical situation of the appendix is described and the method by which foreign material finds its way into it is thus detailed:

"Its lumen communicating with the great pouch, the largest part of the alimentary canal, into which is being poured during a good portion of the time, semi-liquid ingested matter, and various undigested particles under considerable pressure, no matter what position the subject assumes, it is no wonder that these contents find their way into this organ. Once in, the tendency is to remain; for this diminutive process has little peristaltic power to empty itself. Hence, by pressure of solid particles or fecal enteroliths formed by partial evaporation of the liquid contents, ulcers are formed, and perforation, more or less rapid and complete, may occur, with escape of gas or other contents, or, without perforation, inflammation may be instituted, which spreads through the walls, and fires up the peritoneum in immediate contact. Moreover, pressure of the overlying and usually filled cecum directly upon the appendix must be an additional factor in the interference with its proper nutrition and the resulting breaking down.

"Again, when distended with gas, or even when empty and small, such is its unfortunate position that this pressure may shut off a proper blood supply and cause gangrene, general or localized pressure is undoubtedly increased in the sitting posture, and this may, in part, account for the frequency of appendicitis in those of sedentary habits."

The symptoms of catarrhal appendicitis and gangrene (general and local) are given, and these forms of appendicitis are illustrated by detailing cases belonging to one or the other type. The same is done with recurrent appendicitis.

The use of the exploring needle in diagnosticating the presence of pus is not justifiable, as it may be followed by serious consequences. "When the symptoms point to pus accumulations, the most surgical procedure is to cut down upon the swelling. If the peritoneum is adherent, you simply enter the abscess cavity. If you enter the peritoneal cavity, the abscess can be recognized and drained through an incision posteriorly and the anterior incision closed. A very small pus sac may be opened, scraped and cleaned, and packed with iodoform gauze without any posterior opening."

The author concludes with a description of the operative technique in the removal of the appendix, as follows :

"An incision about five inches long is made through the abdominal wall, parallel with the linea alba. The center of this incision should be about one-half of an inch below a line drawn from the anterior superior spine of the ilium to the umbilicus. All bleeding should be stopped by ligature before the peritoneum is opened. When this is done, any loops of small intestine which may protrude should be gently pushed toward the median line and held away by sterilized gauze napkins. The cecum is readily seen, and may be recognized by the longitudinal band, which, if followed downward, leads directly to the appendix. Should no adhesions be found, the end of the cecum may be slightly lifted and thus bring the appendix into view, when it can be dissected from its peritoneal attachments, commencing at the apex. About onefourth of an inch from its junction with the large intestine, a strong silk ligature is applied around it, and the appendix cut away with the scissors from one-eighth to one-fourth of an inch beyond the ligature. The end of the stump is rendered aseptic by thoroughly applying over its cut a surface solid tablet of mercuric bichloride. The ends of the ligature are cut off close to the knot after the toilet of the peritoneum. A piece of rubber tissue protective, disinfected by immersion in 1-5000 cool bichloride solution, is now placed so as to wall off the small intestines from the stump, and against this tissue is placed the iodoform gauze which fills the wound and projects to the surface through its lower one or two inches which is not closed by sutures. The rubber tissue is used because it does not adhere to the intestines as does the gauze. The upper remaining portion of the wound is closed as after an ordinary laparatomy. This dressing may remain for from three to ten days, preferably about seven days, when the gauze and tissue protective are pulled out, the wound irrigated with 1-3000 bichloride solution. A small strip of iodoform gauze or a soft rubber tube may be carried down to the bottom of the wound to insure drainage, and this finally removed in another week or ten days. For fear of ventral hernia, the patient should keep in bed for four or six weeks, to permit the lower part of the wound to close by strong cicatricial adhesion.

"When adhesions between contiguous loops of intestine and the appendix or cecum are met with, these should be carefully separated, and upon the discovery of pus or extravasated matter, this should be mopped out with sponges, taking care to prevent, if possible, the contact of septic matter with the general peritoneal cavity. The use of sterilized gauze napkins may aid in walling off the general cavity.

When the appendix is gangrenous or greatly distended, or has already been perforated, very considerable care is essential in dissecting it loose, either to prevent rupture or to avoid the escape of its contents.

"When a large agglutination is encountered, and the presence of a larger quantity of collected pus is evident, it is safer to use the anterior incision, which led to this discovery, as a guide to the entrance of the abscess from a posterior and retro-peritoneal incision through which drainage can be effected. The anterior wound can then be closed throughout by sutures; if not contra-indicated, I prefer good sterilized silk for the sutures passing through the skin, and carefully going through each layer of fascia and muscle, and the abdominal peritoneum at least one fourth of an inch from the cut edge of the peritoneum. When a general toilet of the peritoneum is indicated, I prefer irrigating this cavity with sterilized water (just boiled and cooled down to 100° F.). This is carried in from an elevated irrigator through a stiff blunt gutta-percha tube (boiled before using). As the general peritoneal cavity becomes filled, the fluid flows out through the opened wound. The remainder is sponged out until the cavity is dry and clean. I have once made a second opening in the median line in order to drain the deep pelvis. This is rarely indicated."

In conclusion upon this subject, Dr. Wyeth says: "Contrary to former teaching, so-called perityphlitis and perityphlitic abscesses are intraperitoneal lesions. They seemed to be retroperitoneal after adhesions had taken place."

